# Cigarette Smoking

**Published:** 1900-08-18

**Authors:** 


### The Hospital

A Journal of

The Medical Sciences and Hospital Administration.

Vol. XXVIII.—No. 725.] Edited by Sir Henry Burdett, K.C.B., and Solomon C. Smith, M.D., M.R.C.P. [August 18, 1900.

### Cigarette Smoking.

We learn from the daily papers that a great American railway company has introduced into its service a rule prohibitory of cigarette smoking, on the ground that indulgence in this habit renders men less trustworthy in the discharge of the duties which devolve upon them, less on the alert with reference to adverse signals or other indications of danger, and less prepared to cope with any emergencies which may befall them than they would be in what, for the purposes of the argument, may be described as their natural condition. A belief to this effect has, we know, long been entertained throughout the whole of what may be called the "Wild West" of the United States; but we are not aware upon what evidence, other than general experience, it is supposed to be founded. Pipes, and even cigars, do not fall under similar condemnation, and it is quite possible that the difference may depend mainly upon the question of quantity. Cigarettes are extremely cheap, extremely portable, and burn quickly, so that a great deal of tobacco may be consumed in a limited time, not to add that there is some temptation to use the stump of each one as a means of kindling its successor, and thus to continue smoking almost without intermission over considerable periods. We have never seen an English railway official smoking on duty, and therefore imagine that the practice is prohibited; but, in the States, it is presumably permitted. Coincidentally with this American "counterblast," we read also that the School Board of Edinburgh is taking action in a parallel direction, and is endeavouring, by visitation, leaflets, prohibitions, and other means, to check the smoking of cigarettes by the rising generation of that city. The Board asserts that the influence of cigarette smoking upon boys is altogether hurtful; that it stunts their growth, diminishes their muscular development, muddles their brains, and corrupts their morals. Here, again, we do not know upon what amount or kind of facts the indictment may be based, but it seems reasonable to believe that the action of a powerful narcotic upon the undeveloped body is not calculated to be beneficial. It is a matter of common knowledge, for example, that opium and its derivatives are much more dangerous, in proportion, to children than to adults; while no one who has witnessed the effect of a tobacco enema, or even that of a boy's first smoke, can question that the extraordinary rapidity with which tolerance of tobacco is acquired is the main cause of our forgetfulness of its essentially posionous character. The whole question is unfortunately liable to be confused by likes and dislikes, and by the errors incidental to generalisation upon limited or even upon individual experience; but it is nevertheless well worthy of impartial examination by scientific methods. The practice of smoking has become an almost universal national indulgence, or as some would say, a prevailing national vice; and it is hardly conceivable that it can be without influence of some sort upon the national capacities and the national character. It should be the business of physiologists to tell us what its influence is, and in what direction and to what extent it is exerted. At present, little is certainly known as to its physical effects, except that excessive smoking is a not uncommon cause of blindness; while as to its "moral" effects, upon which the Edinburgh School Board is said to lay stress, few impartial observers will deny that the average uncultivated young man, the clerk, or messenger, or shopboy, is impelled by smoking into excesses of selfishness and ill-manners to which he would hardly otherwise have attained. He puffs his smoke into the faces of ladies on railway platforms, in the streets, or on omnibuses; he scatters lighted matches in unrestrained profusion, and he throws the still burning stump of his cigarette upon the back of a passing horse or upon any handy inflammable material. To him, whatever may be the real and undeniable uses of tobacco to adults exposed to hardships or engaged in work, his smoking is nothing but an absolutely wasteful, selfish, and useless indulgence, by which he cannot by any possibility derive anything conducive to his permanent advantage. The one cogent argument in favour of the process is that smokers contribute something like twelve millions sterling annually to the national exchequer. The practice of excessive drinking is clearly upon the decline, but it is equally clear that the practice of smoking is increasing in at least an equal proportion. A somewhat comically composed committee of teetotallers is said to be engaged in "investigating," save the mark, the influence of heredity in conducing to intemperance; and they might manifestly widen the scope of their inquiry. The pendulum of English taste seems at present to be swinging away from alcohol, and especially from alcoholic excess, and it would certainly be worth our while to ascertain precisely what it is likely to find at the other extremity of its arc of movement.

